# Chloroplast genome comparison of *Valeriana* species with sequence variation, selective pressure, and divergence analysis

**DOI:** 10.1371/journal.pone.0344868

**Published:** 2026-03-17

**Authors:** Sumin Jeong, Yong-Goo Kim, Yeseul Kim, Bo-Mi Nam, Yun Ji Park, Byung Jun Jin, Inkyu Park

**Affiliations:** 1 Department of Biology, Changwon National University, Changwon, Republic of Korea; 2 Department of Herbal Crop Research, National Institute of Horticultural & Herbal Science, RDA, Eumseong, Republic of Korea; 3 Division of Botany, Honam National Institute of Biological Resources, Mokpo, Republic of Korea; 4 Global advanced Institute of Nano Technology, Changwon National University, Changwon, Republic of Korea; Nuclear Science and Technology Research Institute, IRAN, ISLAMIC REPUBLIC OF

## Abstract

*Valeriana fauriei* and *V. dageletiana* are traditional medicinal plants known for their calming effects and use in alleviating insomnia. However, genomic information for these species is limited. This study aimed to sequence and characterize the complete chloroplast genomes of *V. fauriei* and *V. dageletiana*, and to compare them with those of closely related *Valeriana* species to investigate structural variation, molecular evolution, and divergence history. The chloroplast genomes of *V. fauriei*, *V. dageletiana*, and *V. jatamansi* were highly conserved in overall structure. Minor differences were observed in tandem repeat regions and sequence divergence hotspots, particularly within *accD*, *rps18*, and the *trnN–trnL* intergenic region. Analysis of 57 protein-coding genes from four species revealed that most genes are under strong purifying selection. However, elevated dN/dS ratios in *psbI*, *rps7*, and *rpl23* suggest potential lineage-specific divergence. Phylogenetic reconstruction showed that *V. fauriei* and *V. dageletiana* form a clade with *V. officinalis*, whereas *V. jatamansi* is an earlier diverging lineage. Divergence time estimation indicated that *V. officinalis* split from this clade 0.4255 to 1,0839 million years ago, and that *V. fauriei* and *V. dageletiana* diverged approximately 0.0205 million years ago. These results provide insights into the evolution of *Valeriana* chloroplast genomes, highlighting both structural conservation and species-specific variation. The findings contribute to a better understanding of recent speciation events and molecular evolution in this genus, supporting future phylogenomic and taxonomic studies of *Valeriana* species.

## Introduction

*Valeriana* L. is a perennial plant belonging to the order Dipsacales and family Caprifoliaceae, with 200–300 species distributed worldwide. In East Asia, the genus *Valeriana* has been used primarily for medicinal purposes. In Korea, *Valeriana fauriei* Briq. and *Valeriana dageletiana* Nakai ex F.Maek. have been particularly noted for medicinal use. Geographically, *V. fauriei* occurs in Korea, northeastern China, Japan, and Sakhalin (Russia), in contrast to *V. dageletiana*, which is endemic to Ulleungdo, a volcanic island in Korea [1–3]. *V. fauriei* and *V. dageletiana* share typical *Valeriana* morphology: erect stems (ca. 40–100 cm) with white hairs concentrated at the nodes; opposite, pinnate leaves divided into 3–7 serrated leaflets; and corymbose inflorescences of light pink flowers, with linear bracts approximately 1 cm long. However, relative to *V. fauriei*, *V. dageletiana* is generally larger, bears broader and more bluish leaves, and has stems that are nearly glabrous except at the nodes, allowing practical differentiation between the two species [4].

The dried roots and rhizomes of *V. fauriei* and *V. dageletiana* have been used as traditional medicinal resources for centuries [5]. These dried materials have been used to treat nervous system conditions, such as mental anxiety, depression, and insomnia [6–8]. They can also be used as antioxidants and to prevent muscle atrophy, and as anti-obesity agents [9,10]. Although pharmacological uses and efficacy have been actively investigated, interspecific relationships, divergence times, and genomic diversity remain insufficiently resolved for many *Valeriana* species. For some groups—including the Ulleungdo endemic *V. dageletiana*—taxonomic treatments are unsettled, leaving their phylogenetic placement and divergence history unclear. This uncertainty impedes efforts to establish standardized comparative baselines for medicinal applications and increases the risk of inconsistency in species identification and authentication. To reduce these gaps, standardized, cross-comparable genomic datasets and a consistent phylogenetic perspective are needed.

The chloroplast (cp) genome is particularly useful for evaluating relationships and divergence among closely related plant taxa [11]. Its genome, typically ranging from 120 to 200 kb, is generally circular or, in rare cases, linear [12]. The cp genome follows a quadripartite structure, consisting of two inverted repeats (IRa and IRb) that separate the small single-copy (SSC) and large single-copy (LSC) regions [13]. Compared to its ancestral form, the current cp genome has undergone significant gene reduction, retaining approximately 80 protein-coding genes, 4 rRNA genes, and 30 tRNA genes [14]. In angiosperms, cp genomes are relatively stable, reflecting their predominantly maternal inheritance, and they serve as a powerful tool for resolving phylogenetic relationships and tracing divergence histories [15].

Despite its stability, the cp genome is not entirely conserved, as genetic variations accumulate over evolutionary timescales. One key approach to understanding these changes is analyzing selection pressures on genes through the dN/dS ratio, which compares the rate of nonsynonymous (dN) to synonymous (dS) substitutions [14]. A ratio >1 indicates positive selection, < 1 suggests purifying selection, and ≈ 1 implies neutral evolution [16–20]. Studies on dN/dS ratios in the context of plant evolution have provided important insights. For example, studies of plant cp genomes have shown that genes involved in photosynthesis often undergo strong purifying selection, reflecting their important roles in plant fitness [21]. Conversely, some cp genes (e.g., *ycf1*, *ycf2*, *accD*, *clpP*, *matK*, and *ndh*) show lineage-specific increases in dN/dS, consistent with episodic positive selection, relaxed purifying selection in lineages with reduced photosynthesis, or cp structural changes that modify selective constraints [22].

Time-calibrated cp phylogenies enable reconstruction of divergence histories and the timing of lineage-specific rate or selection shifts. This approach provides a chronological basis for assessing the effects of selection on cp genome variation [23]. In a previous study, Shrestha et al. (2019) constructed a cp phylogeny and revealed lineage-specific rate accelerations and selection signals in photosynthesis-related genes of *Passiflora* [24]. Gao et al. (2019) similarly detected positive selection in photosynthesis-related genes of *Oryza*, implicating adaptation to light environments [25]. In Campanulaceae, Li et al. (2020) identified numerous selected sites across Cyanantheae lineages, which also clarified intrafamilial relationships [26]. Together, these studies show that although cp genes are generally under purifying selection, they also reveal lineage- and gene-specific episodes of adaptive evolution, indicating when and where selection has shaped the cp genome.

In this study, we aimed to resolve interspecific relationships and estimate divergence times for the East Asian *Valeriana* species examined here by characterizing sequence and structural variation in cp genomes and applying time-calibrated cp phylogenies. We further aimed to assess evolutionary dynamics at both the genic and phylogenetic levels to better understand patterns of lineage diversification. These objectives provide a basis for future comparative studies incorporating nuclear genomic data and can inform molecular discrimination and quality assessment of closely related medicinal taxa, including *V. fauriei* and *V. dageletiana*.

## Materials and methods

### Plant material

Fresh *V. fauriei* leaves were collected from cultivation field at the Department of Herbal Crop Research, National Institute of Horticultural & Herbal Science and *V. dageletiana* leaves were collected from their natural habitats in South Korea. The leaves were dried and registered as specimens at HNIBRVP18373. Information regarding the collection locations and voucher numbers of the specimens used in this study are presented in S1 Table. Collection permission was granted by the appropriate local authorities for the collection of wild plant materials. These samples were identified by Dr. Bo-Mi Nam and deposited at the Division of Botany, Honam National Institute of Biological Resources.

### Chloroplast genome sequencing and assembly

We extracted total genomic DNA using the modified cetyltrimethylammonium bromide protocol of Allen et al. (2006) [27], which is optimized for medicinal plants rich in secondary metabolites and yields high-purity DNA suitable for next-generation sequencing. Illumina libraries were prepared from total DNA, and paired-end reads were generated using a NovaSeq 6000 platform (Illumina, San Diego, CA, USA). Reads obtained through sequencing were quality-checked and trimmed using Trimmomatic ver. 0.39 [28]. Trimmed reads (Phred quality score ≥ 20) were assembled with NOVOplasty ver. 4.3.3 [29] using default parameters. Based on the aligned paired-end reads, gaps were filled using SOAPdenovo GapCloser [30]. Finally, the trimmed paired-end reads were mapped to whole-genome sequences using BWA ver. 0.7.17 [31].

### Repeat analysis

Using MISA [32], simple sequence repeats (SSRs) containing mono-, di-, tri-, tetra-, penta-, and hexanucleotides were detected, with minimum numbers set at 10, 5, 4, 3, and 3. Tandem repeats were identified using Tandem Repeats Finder [33], with the minimum alignment score and maximum period size set at 50 and 500, respectively. Forward, reverse, palindromic, and complementary repeats were detected using REPuter [34].

### Genome annotation and comparative analysis

The circular genomes of the two *Valeriana* species were annotated using Geseq [35]. The tRNAs were identified using tRNAscan-SE ver. 1.21 [36]. Based on the cp genome annotation results, a cp genome map was constructed using OrganellarGenomeDRAW (OGDRAW) ver. 1.3.1 [37]. The GC content and codon usage of *V. fauriei* and *V. dageletiana* were analyzed using MEGA11 [38]. The codon usage distribution of the cp genomes was visualized using the Heatmapper program and hierarchical clustering method. The *V. fauriei*, *V. dageletiana, V. officinalis*, and *V. jatamansi* cp genomes were compared using the mVISTA program [39] in Shuffle-LAGAN mode. Nucleotide diversity (Pi) values were calculated using DnaSP ver.6 [40].

### Selection pressure analysis

To perform the dN/dS analysis, we first used Geneious software [41] (Biomatters, Auckland, New Zealand) to align and extract 57 protein-coding genes from each of the four *Valeriana* species (*V. fauriei*, *V. dageletiana*, *V. officinalis*, and *V. jatamansi*) using *F. cornucopiae* as the reference genome. Interspecific genetic distances for tree file generation were calculated based on the Kimura 2-parameter (K2P) model in MEGA11 [38]. 2021. Next, we estimated the dN/dS value for each protein-coding gene using the CODEML program in Phylogenetic Analysis by Maximum Likelihood (PAML) v.4.9 [42]. In our PAML analysis, we determined codon frequencies using the F3 × 4 model, which considers the frequency of each nucleotide at three codon positions. We used the M1a (nearly neutral) and M2a (positive selection) models to compute the dN and dS ratios and conduct the likelihood ratio test (LRT). By comparing the log-likelihoods of the M1a and M2a models, we compared the fit of the two models using the LRT and assessed whether positive selection was significant in the M2a model. To visualize the distribution of dN/dS values across the four *Valeriana* species, box plots were constructed using the R package ggplot2 [43].

### Phylogenetic analysis and divergence time estimation based on chloroplast genome sequences

In this study, 9 cp sequences, including those of *V. fauriei* and *V. dageletiana,* were used for phylogenetic analysis. The sequences for all taxa—excluding the two *Valeriana* species assembled in this study—were downloaded from NCBI; their accession numbers are listed in S2 Table. The cp genome was aligned using Multiple Alignment using Fast Fourier Transform (MAFFT) ver. 7 [44] and 57 coding sequences (CDSs) were extracted from the aligned sequences using Geneious software [39]. CDS datasets were filtered to remove ambiguously aligned regions using GBlock ver. 5 [45].

Using jModelTest ver. 2.1.10 [46], we selected the GTR + I + G model for ML and BI analyses. ML analysis was performed using MEGA11 [38] with 1,000 bootstrap replications. BI analysis was performed using MrBayes ver. 3.2.6 [47] with the Markov chain Monte Carlo (MCMC) algorithm, running for 5,500,000 generations and utilizing two independent runs of two simultaneous chains. Phylogenetic trees were sampled every 4,000 generations, and the initial 10% were discarded as burn-ins. Trees were constructed according to a 50% majority consensus to estimate posterior probabilities.

To estimate the divergence time of Valerianeae, Bayesian Evolutionary Analysis Sampling Trees (BEAST) ver. 1.856 [48] was used based on 66 CDSs extracted from nine aligned cp genome sequences, including *D. japonicus*, *D. asper*, *S. tschiliensis*, and *S. comosa*. Input files were prepared in the Bayesian Evolutionary Analysis Utility (BEAUti) interface to configure the parameters required for phylogenetic analysis. The general time reversible (GTR) model was selected as the nucleotide substitution model, and the Yule tree prior was used to model species diversification. Analyses were run under a strict molecular clock, which provided markedly improved mixing and stable convergence relative to relaxed-clock runs in this dataset of nine taxa with a single secondary calibration. The Valerianoideae–Dipsacoideae split (MRCA of the two subfamilies) was calibrated using a secondary calibration from Wang et al. (2020): 70.19 Ma with a 95% HPD of 51.23–92.43 Ma [49]. To avoid imposing additional distributional assumptions, a Uniform prior U (51.23, 92.43) Ma was applied to this node. Monophyly constraints were enforced on Valerianoideae and Dipsacoideae to prevent calibration distortion due to topology changes. MCMC chains were run for 10 million generations with a burn-in of 10% and sampling every 1,000 generations. Stationarity and mixing were assessed in Tracer ver. 1.7 [50] by inspecting trace plots and posterior densities, verifying stable convergence across key parameters. Posterior trees were summarized as a maximum clade credibility tree in TreeAnnotator ver. 1.8 with a posterior probability limit of 0.50. Mean node heights and 95% HPD intervals were reported from TreeAnnotator outputs, and results were visualized in FigTree ver. 1.4.2 [51] to provide a clear representation of the evolutionary relationships and divergence times among Valerianeae species.

## Results

### Chloroplast genome characterization and *Valeriana* genetic variation

**Fig 1:**
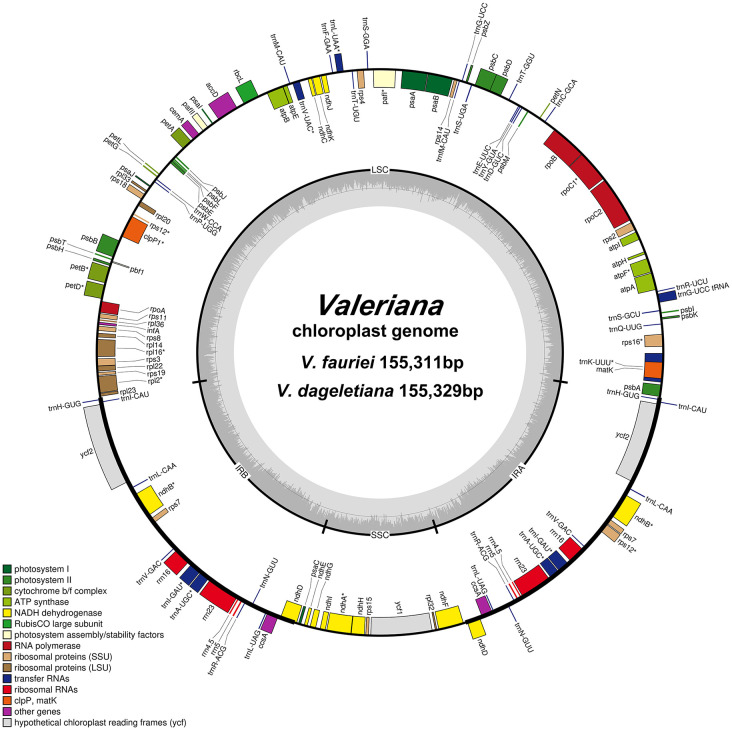
The circular chloroplast genome maps of *V. fauriei* and *V. dageletiana.* Genes inside the circle are clockwise, and genes outside the circle are counterclockwise. The dark gray graph inside the circle shows GC%. The thick lines in the large circle represent the two inverted repeat (IR) regions dividing the large single-copy (LSC) and small single-copy (SSC) regions. Label intron-containing genes with asterisks.

The cp genomes of *V. fauriei* and *V. dageletiana* were sequenced with coverages of 1,328× and 453 × , respectively (S3 Table). We generated 12.7 and 12.3 Gb paired-end reads, resulting in 5.5 and 9.7 Gb of trimmed reads, respectively, with the sequencing platform and raw read data provided in S4 Table. The coverage and read depth of the assembled genome are shown in S1 Fig. The cp genomes of *V. fauriei* and *V. dageletiana* have circular structures with LSC and SSC regions separated by two IR regions (Fig 1). The overall genome characteristics of *V. fauriei* and *V. dageletiana* were as follows (Table 1): The lengths of the cp genomes were 155,329 bp for *V. fauriei* and 155,311 bp for *V. dageletiana*. The length ranges of each region were 85,478–85,568 bp for LSC, 15,159–15,195 bp for SSC, and 27,301–27,319 bp for IR. The GC contents of the LSC, SSC, and IR regions were 36.7, 32.6, and 42.5%, respectively, identical in both *Valeriana* species. The cp genome contained 113 unique genes comprising 79 protein-coding, 4 ribosomal RNA, and 30 transfer RNA genes. Seventeen genes had introns, of which four (*ndhB*, *rps12*, *trnA-UGC*, and *trnI-GAU*) were located in the IR region and had duplicate copies (S5 Table). Additionally, 14 genes had a single intron, while *pafI* and *clpP1* had two introns (S6 Table).

**Table 1:** Characterization of *V. fauriei* and *V. dageletiana* chloroplast genomes.

Characteristic	*V. fauriei*	*V. dageletiana*
Accession number	PQ280048.1	PQ280047.1
**Genome size**		
Total cp genome (bp)	155,329	155,311
Large single-copy (LSC) region (bp)	85,568	85,478
Inverted repeat (IR) region (bp)	27,301	27,319
Small single copy (SSC) region (bp)	15,159	15,195
**Number of unique genes**		
Total	113	113
Protein-coding genes	79	79
rRNA genes	4	4
tRNA genes	30	30
**GC content (%)**		
Total genome	38.4	38.3
LSC region	36.7	36.7
IR regions	42.5	42.5
SSC region	32.6	32.6

A total of 27,087 and 27,079 codons were detected in *V. fauriei* and *V. dageletiana*, respectively (S2 Fig). The most frequently detected codons in both cp genomes were leucine, isoleucine, and serine. Analysis of relative synonymous codon usage (RSCU) values showed that arginine, leucine, and serine had the highest RSCU values among the 20 amino acids and stop codons. Codon pattern analysis revealed that the codon usage bias of the two *Valeriana* species showed similar overall patterns. In S3 Fig, green represents a strong codon bias (RSCU > 1), indicating frequent usage of specific codons, while red represents a weak codon bias (RSCU < 1), indicating less frequent usage.

**Fig 2:**
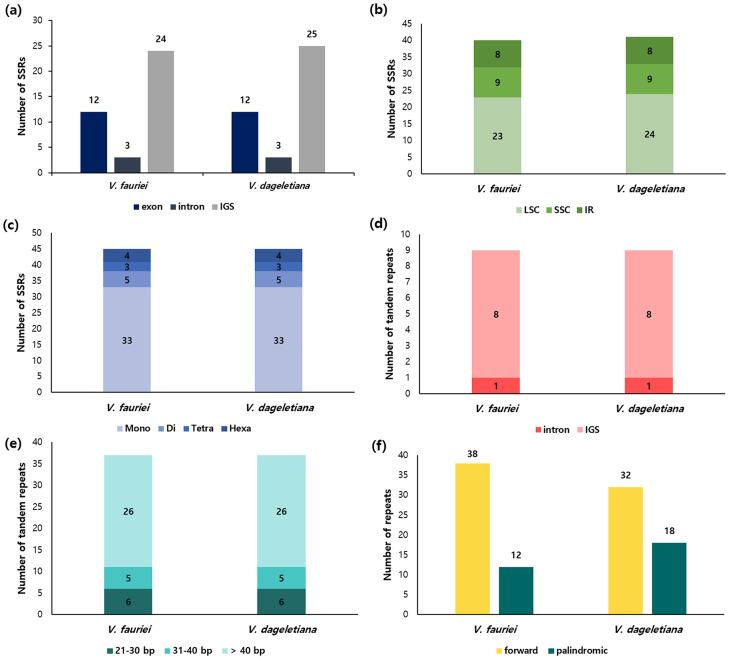
Repeat sequence analysis of *V. fauriei* and *V. dageletiana.* **(a)** Number of single sequence repeats (SSRs) in exons, introns, and intergenic spacers (IGSs). **(b)** Number of SSRs present in large single-copy (LSC) and small single-copy (SSC), and inverted repeat (IR) regions. **(c)** Frequency of the four different SSR types. **(d)** Number of tandem repeats present in exons, introns, and IGSs. **(e)** Distribution according to the length of tandem repeats. **(f)** Frequency of different repeat types in the chloroplast genome.

The number and characteristics of SSRs and tandem repeats, as well as the different patterns appearing in the genome sequence, were examined in the *Valeriana* cp genome (Fig 2). Twenty-nine and 30 SSRs were identified in *V. fauriei* and *V. dageletiana*, respectively, with the highest number detected in the intergenic spacers (IGSs) (Fig 2a). SSRs were most frequently found in the LSC region and predominantly comprised mononucleotides (Fig 2b, 2c). Regarding tandem repeats, 18 were found in the IGSs, and 1 was found in the intron. These were identical in both *Valeriana* cp genomes (Fig 2d). The number of tandem repeats by size category was also identical in both species, with 6 repeats of 21–30 bp, 5 of 31–40 bp, and 26 greater than 40 bp (Fig 2e). The forward and palindromic repetition sequences were 38 and 12 in *V. fauriei* and 32 and 18 in *V. dageletiana* (Fig 2f).

### Comprehensive comparative analysis of *Valeriana* chloroplast genomes

To understand the structural changes contributing to genomic diversity among species, the contraction and expansion of the *Valeriana* IR region in the three species were compared with those in *Fedia cornucopiae* (S4 Fig). A comparison of the lengths of the four cp genome regions revealed that slight IR expansion occurred in the three *Valeriana* species. Compared with *F. cornucopiae*, the IR region expanded from 355 to 632 bp, causing the LSC region to contract from 2,518–2,986 bp and the SSC region from 667 to 754 bp. In addition, when comparing the genes located at the boundaries of the cp genome regions, *ndhD*, *psaC*, and *ndhF* located in the IR (IRa and IRb) and SSC regions of *V. fauriei* and *V. dageletiana* had the same locations, which were very similar to those of *V. jatamansi*. The *trnH* of *F. cornucopiae*, located in the IR region, was farthest (203 bp) from the LSC/IR boundary, whereas that of *V. dageletiana* was closest (93 bp). In contrast, in the LSC region, *rpl23* and *psbA* in *V. dageletiana* were farthest from the boundary (113 and 111 bp, respectively). Overall, the *Valeriana* cp genome was highly conserved, and an IR expansion was observed in the three *Valeriana* species relative to *F. cornucopiae*.

**Fig 3:**
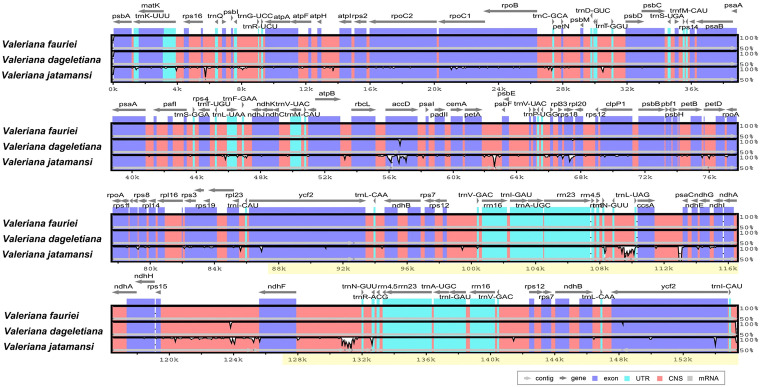
Comparison of the chloroplast genomes of three *Valeriana* species using mVISTA. Regions where sequence variation exists between chloroplast genomes are shown in white, and identity is indicated in the 50–100% range. Gray arrows above the alignment indicate the forward and reverse orientation of genes. The purple bars represent exons, and the pink bars represent conserved non-coding sequences. The yellow mark below the bar indicates the inverted repeat regions (IRs).

To compare and visualize the genomic divergent regions, mVISTA analysis was performed using *V. fauriei*, *V. dageletiana*, and *V. jatamansi* (Fig 3). *V. fauriei* cp genome sequence alignment revealed the sequence divergence of *accD*, *rps18*, *rps15*-*ndhF*, and *ycf2* from that of *V. dageletiana*. Most sequence variations were found in *V. jatamansi*, and sequence divergence was identified in *accD*, *petA*-*psbF*, *rps18*, *trnN*-*GUU*-*trnL*-*UAG*, *ccsA*-*psaC*, *rps15*-*ndhF*, *ndhF*-*trnN*-*GUU*, and *ycf2* genes.

**Fig 4:**
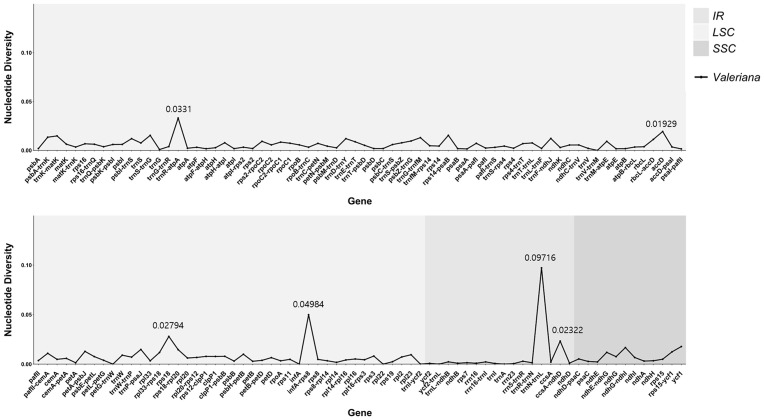
Comparison of the three *Valeriana* species’ nucleotide diversity (Pi) values. The light gray background indicates a large single-copy (LSC) region, gray indicates an inverted repeat (IR) region, and dark gray indicates a small single-copy (SSC) region. The top six values in the data are indicated numerically in the figure.

To confirm that sequence divergence occurred in *Valeriana*, we calculated the nucleotide diversity (Pi) values of genes and IGSs in *V. fauriei*, *V. dageletiana*, and *V. jatamansi* (Fig 4). The regions with high Pi values in the three species were *trnN-trnL* (0.09716), *infA-rps8* (0.04984), *trnR-atpA* (0.0331), *rps18* (0.02794), *ccsA-ndhD* (0.02322), and *accD* (0.01929). The lowest Pi value excluding Pi = 0 was *rrn23* (0.00047). Most Pi values in *Valeriana* were low, but notable hotspots were found in the LSC and IR regions.

### Selection analysis

**Fig 5:**
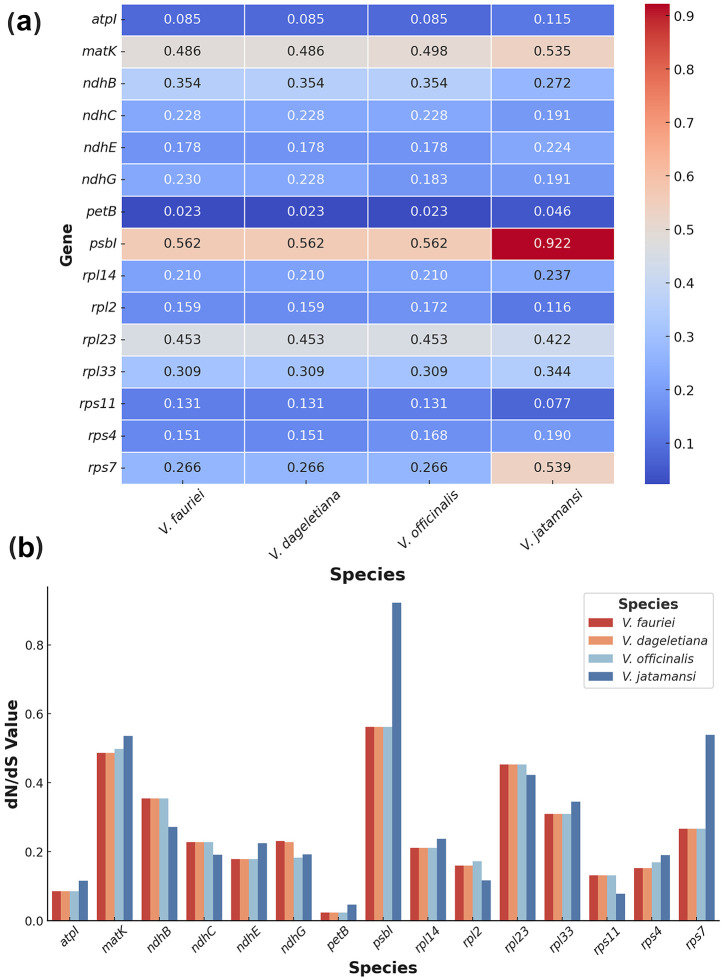
dN/dS ratios of chloroplast protein-coding genes across *Valeriana* species. (a) Heatmap illustrating the dN/dS values for 14 selected protein-coding genes in *V. fauriei*, *V. dageletiana*, *V. officinalis*, and *V. jatamansi*. The color gradient represents dN/dS values, with blue indicating lower ratios and red indicating higher ratios. (b) Bar chart showing the same dN/dS values for these genes across the four species, with different colors representing each species.

In our study, we used *F. cornucopiae* as the reference genome to calculate the dN/dS values for the cp genes of *V. fauriei*, *V. dageletiana*, *V. officinalis*, and *V. jatamansi* (Fig 5). To assess selection patterns among species, we applied two site models: M1a (nearly neutral) and M2a (positive selection), and the results are summarized in S7 Table. Likelihood Ratio Tests (LRTs) were performed to compare the two models (S8 Table), and the results indicated that M1a was the most appropriate model (*p* > 0.05) in most cases. Negative or purifying selection was the predominant evolutionary force acting on cp genes in *Valeriana* species. Consequently, the M1a model results were used for further analyses.

The dN/dS values ranged from 0.023 to 0.9224 across *V. fauriei*, *V. dageletiana*, *V. officinalis*, and *V. jatamansi*, with interspecies differences in selection pressure. Most genes exhibited low dN/dS ratios, consistent with strong purifying selection, while a subset showed relatively higher values. Among them, *psbI* (0.9224) in *V. jatamansi* displayed particularly elevated values. In contrast, *petB* showed consistently low ratios across species (0.023–0.046). LRT results further supported these patterns, as most genes showed non-significant differences between M1a and M2a (*p* > 0.05), confirming that purifying selection predominates across *Valeriana* cp genes. While most genes were highly conserved, interspecies variation in selection pressure was observed, with *V. jatamansi* displaying the most pronounced differences.

### Molecular dating of *Valeriana*

**Fig 6:**
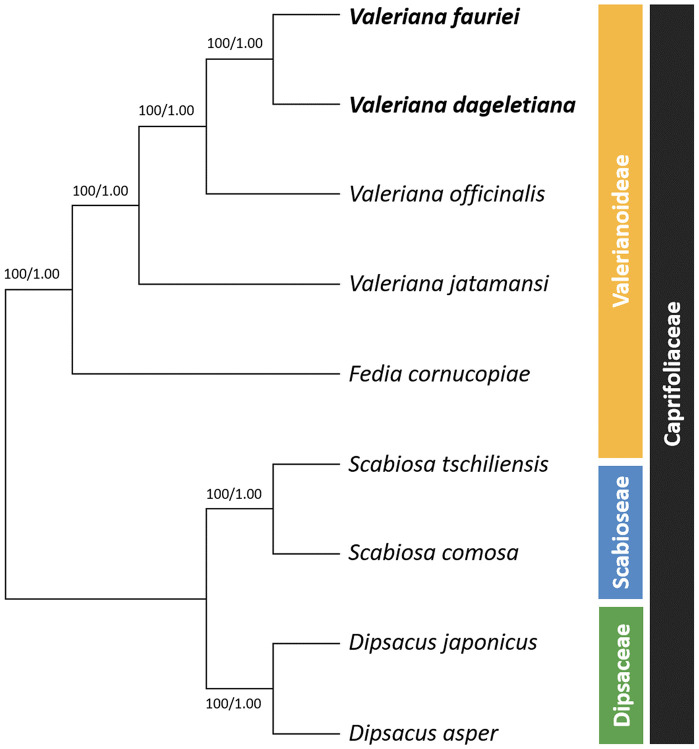
Phylogenetic tree showing the combined Maximum Likelihood (ML) bootstrap and Bayesian Inference (BI) probabilities based on 66 coding sequences (CDSs) of *Valeriana*, with *F. cornucopiae* as the closest relative and Dipsacaceae and Scabioseae as the outgroups. Numbers at the nodes indicate ML bootstrap (left) and BI posterior probabilities (right).

To determine the phylogenetic relationships among *Valeriana*, a phylogenetic tree was constructed using 66 CDSs from nine species, including *Valeriana*, *Fedia*, *Scabiosa*, and *Dipsacus* (Fig 6). The phylogenetic tree showed strong support across all relationships (ML = 100%; BI = 1.00), and the analyses confirmed that *V. dageletiana* is closely related to *V. fauriei*. Additionally, the analysis revealed that *V. jatamansi* was the first to diverge within *Valeriana*, followed by *V. officinalis*. Subsequently, *V. fauriei* and *V. dageletiana* formed a strongly supported clade, indicating a closer evolutionary relationship between these two species. This phylogenetic pattern suggests that while *V. jatamansi* and *V. officinalis* represent earlier diverging lineages, *V. fauriei* and *V. dageletiana* share a more recent common ancestor. Overall, *V. dageletiana* and *V. fauriei* are more closely related to *V. officinalis* than *V. jatamansi*.

**Fig 7:**
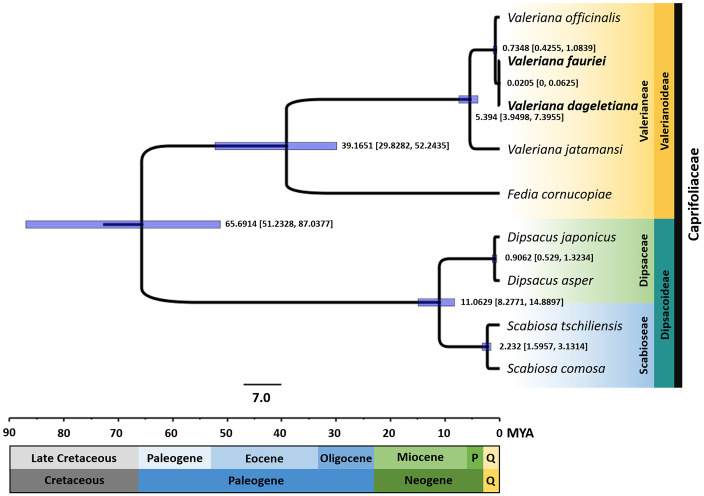
Divergence time estimates based on a Bayesian tree using 66 protein-coding genes. Operational taxonomic units (OTUs) represent Valerianeae, Dipsacaceae, and Scabioseae. Numbers along branches denote mean divergence ages (million years ago, MYA), and 95% highest posterior density (HPD) intervals are shown for each node. P: Pliocene; Q: Quaternary (includes the Holocene and Pleistocene).

Our phylogenetic analysis also provided detailed estimates of divergence times among *Valeriana* species and related families (Fig 7). The divergence between Valerianeae and the clade comprising Dipsaceae and Scabioseae is estimated at approximately 51.2328–87.0377 Mya, marking a deep split in their evolutionary history. Within *Valeriana*, *F. cornucopiae* separated from the lineage around 29.8282–52.2435 Mya, indicating an early divergence event. Approximately 3.9497–7.3955 Mya, *V. jatamansi* diverged from a common ancestor shared with *V. officinalis*, *V. fauriei*, and *V. dageletiana*, representing one of the earliest splits within the genus. Subsequently, *V. officinalis* split from the clade containing *V. fauriei* and *V. dageletiana* around 0.4255–1.0839 Mya, further refining the group’s evolutionary trajectory. The most recent speciation event occurred between *V. fauriei* and *V. dageletiana*, which diverged only 0–0.0625 Mya, indicating an extremely recent evolutionary shift.

## Discussion

We determined the complete cp genomes of *V. fauriei* and *V. dageletiana* in the present study. The lengths of the complete cp genomes were 155,311 bp for *V. fauriei* and 155,329 bp for *V. dageletiana*. The *Valeriana* cp genome had a quadripartite structure with LSC, SSC, and two IR regions. The total GC contents of *V. fauriei* and *V. dageletiana* were 38.4 and 38.3%, respectively, similar at 36.7% for LSC, 32.6% for SSC, and 42.5% for IR. As expected, the GC content in the IR region was higher than in the two single-copy regions [52]. The two cp genomes contained 113 unique genes, including 79 protein-coding, 4 rRNA, and 30 tRNA genes. Their genome length, structure, GC content, and gene number were consistent with previously described angiosperm cp genomes [53]. Codon usage has been closely linked to accurate and efficient gene expression and cp evolutionary history [54–56]. *V. fauriei* and *V. dageletiana* had almost identical codon numbers and RSCUs, indicating synonymous codon usage bias, which was also similar between the two *Valeriana* species. Analysis of RSCU values showed that half had a strong codon bias (RSCU > 1), and codons with high RSCU values had A or T at the third amino acid position. The RSCU values were consistent with the pattern in seed plants, and RSCUs with high A or T ratios at the third position appeared to be similar to those of other cp genomes [57]. CGC (arginine), CTC (leucine), and CTG (leucine) had relatively low RSCU values, whereas GCT (alanine), TAA (termination), and AGA (arginine) had high RSCU values, indicating that they are relatively more conserved amino acids.

SSRs distributed in a genome are useful for analyzing genetic diversity, identifying molecular markers and genes, and identifying genetic relationships between species [58–60]. The SSRs distributed throughout the cp genome were the same in both *Valeriana* species, and the number of mononucleotides was the largest. The *Valeriana* cp genome did not contain tri- and pentanucleotides but five, three, and four di-, tetra-, and hexanucleotides, respectively. The largest number was detected in IGSs (24–25), followed by exons (12) and introns (3). Additionally, SSRs were the highest in the LSC region (23–24), whereas those in the SSC (9) and IR (8) regions were similar. The largest number of SSRs was observed in IGSs because most repeats in the cp genome were richer in A and T repeats than with C and G repeats [61,62]. In the genomes of the two *Valeriana* species, 29–40 tandem repeats were detected, with the highest number of tandem repeats in *V. dageletiana*. Variations in tandem repeats in the cp genomes of angiosperms are common and either alter the length of the genome or cause changes in gene expression [63,64].

The cp genome of angiosperms contains genes essential for photosynthesis, and its structure and gene arrangement are relatively well conserved [9,65]. However, the contraction and expansion of IR regions directly affect cp genome length and can also influence the functional stability of the genome and gene expression [66,67]. Understanding these changes will provide important clues for elucidating the cp genome’s evolutionary history and functional mechanisms.

The mVISTA results showed that the cp genome of *Valeriana* is highly conserved, with genic regions being more conserved than the IGS regions. This observation is common in the cp genomes of angiosperms because the genic regions of the cp genome play functionally important roles [53,68]. In the comparison of the cp gene sequences of the three species of *Valeriana*, the *accD*, *petA-psbF*, *rps18*, *trnN-GUU-trnL-UAG*, *ccsA-psaC*, *rps15-ndhF*, *ndhF-trnN-GUU*, and *ycf2* regions were observed as hotspots of genetic variation. These variations reflect genetic differences between species or individuals and may indicate evolutionary changes or environmental adaptations of the cp genome [69,25]. In addition, we obtained Pi values for *V. fauriei*, *V. dageletiana,* and *V. jatamansi*. *Valeriana* has high Pi values in genes and IGSs, such as *trnN-trnL*, *infA-rps8*, *trnR-atpA*, *rps18*, *ccsA-ndhD*, and *accD*, which are mainly located in the LSC and IR regions and may have been acquired through various selection pressures and genomic recombination events related to environmental adaptation [53,70,71]. Hotspot regions with concentrated genetic variations are useful for distinguishing species and genera, and these genetic differences can contribute to solving taxonomic problems [72,73]. In addition, these hotspots could be used as potential markers for species identification [74].

Analysis of the dN/dS ratios of cp genes across *Valeriana* species revealed that most cp genes are subject to strong purifying selection, maintaining their essential functions in photosynthesis and cellular processes. None of the analyzed genes had a dN/dS ratio exceeding 1, indicating an absence of strong positive selection. This pattern is consistent with previous studies demonstrating that cp genes are generally conserved due to their critical roles in photosynthetic efficiency and organelle function [75,76]. However, some genes exhibited comparatively higher dN/dS values, suggesting potential lineage-specific functional divergence rather than adaptive evolution. Among these, *psbI* exhibited the highest dN/dS ratio (0.9224) in *V. jatamansi*. *psbI* encodes a small transmembrane protein that plays a key role in Photosystem II (PSII) stabilization and repair, facilitating efficient electron transport [77]. Comparative analyses of cp genomes in tribe Selineae similarly identified *psbI* among genes with dN/dS > 1 in some lineages, suggesting potential functional divergence [78]. The relatively elevated dN/dS value of *psbI* in *V. jatamansi* may reflect species-specific modifications in response to changes in light availability or environmental pressures, rather than strong positive selection [79]. Similarly, *rps7* and *rpl23*, encoding ribosomal proteins essential for cp translation, exhibited slightly higher dN/dS values in *V. jatamansi* (0.5388) and in *V. fauriei* and *V. officinalis*, respectively. Although these values remain well below 1, indicating that purifying selection is still the dominant force, the relatively higher ratios compared to other species suggest minor lineage-specific modifications in translational regulation [80]. Ribosomal proteins, including *rps7* and *rpl23*, are known to experience occasional functional divergence in response to shifts in translational demand and cp genome architecture [81]. These variations are more likely to reflect fine-tuning of ribosomal function rather than strong adaptive evolution.

Conversely, several genes exhibited exceptionally low dN/dS values, reinforcing their high degree of evolutionary conservation. Among them, *psaA*, *psbC*, and *petA*, which encode core components of Photosystem I (PSI) and PSII, displayed the lowest dN/dS ratios across all species. *psaA*, encoding a subunit of PSI involved in light-driven electron transport, exhibited dN/dS values as low as 0.011, reinforcing its strict functional constraints [82]. Similarly, *psbC*, a key PSII component, and *petA*, encoding the cytochrome f subunit in the cytochrome b6f complex, exhibited strong signatures of purifying selection, consistent with their essential roles in photosynthetic energy conversion [83]. These findings indicate that while cp genes in *Valeriana* remain highly conserved under purifying selection, certain genes—particularly *psbI* in *V. jatamansi*—show species-specific functional modifications, likely influenced by environmental factors and lineage-specific adaptations. The relatively increased dN/dS values of *rps7* and *rpl23* suggest potential adjustments in translational regulation, though their values remain within the expected range for conserved genes. Meanwhile, the extreme conservation of *psaA*, *psbC*, and *petA* highlights their fundamental importance in cp function and their evolutionary stability across plant species.

Complete cp genomes provide sufficient information for studying phylogenetic relationships among plants, including those at low taxonomic levels and unresolved taxa [53,84]. Therefore, we used the ML and BI methods [36,45], which are useful for analyzing the phylogenetic relationships of plants, using a CDS dataset to determine the positions of species within Valerianoideae. The phylogenetic analysis confirmed that *V. fauriei* and *V. dageletiana* formed a sister group, while together with *V. officinalis* and *V. jatamansi*, they constituted a monophyletic clade within *Valeriana*. Among them, *F. cornucopiae* was the first to diverge, followed by *V. jatamansi*. Subsequently, *V. officinalis* branched off, leaving *V. fauriei* and *V. dageletiana* as the most recently diverged sister species. The clustering of *V. fauriei* and *V. officinalis* was consistent with previous ITS data and ML/BI trees that combined *trnL*, *matK-intron*, and *psbA* [85], as well as an ML tree using *accD*, *matK*, *ndhJ*, *psbM–trnD*, *rpoC1*, *trnK intron*, *trnL* IGS, *trnG*, and *ycf5* together with ITS [47].

In addition, we provided detailed estimates of the divergence times between *Valeriana* species and their related families, offering valuable insights into their evolutionary history. Our phylogenetic results showed that the divergence between the Valerianeae clade and Dipsaceae and Scabioseae occurred approximately 65.691 Mya (51.233–87.038 Mya), during the early Paleocene. Subsequently, the split between *F. cornucopiae* and *Valeriana* occurred around 39.165 Mya (29.828–52.244 Mya) in the late Eocene, during a long-term cooling trend that culminated in the Eocene–Oligocene Transition (~34 Mya) and the onset of major Antarctic glaciation [86]. Within *Valeriana*, *V. jatamansi* was the first species to diverge, around 5.394 Mya (3.950–7.400 Mya), near the Miocene–Pliocene boundary. Near this boundary, climate shifted toward an early–middle Pliocene warm interval, with evidence for regional ocean–atmosphere reorganization and altered precipitation regimes; such shifts may have modulated habitat availability and dispersal routes, influencing range dynamics and subsequent divergence in *Valeriana* [87]. Later, *V. officinalis* diverged from the lineage containing *V. fauriei* and *V. dageletiana* approximately 0.735 Mya (0.426–1.084 Mya), during the middle Pleistocene. The most recent speciation event occurred between *V. fauriei* and *V. dageletiana*, which diverged only 0.021 Mya (0–0.063 Mya), making this a very recent evolutionary event likely influenced by climatic oscillations of the Last Glacial Period. Consistent with other East Asian mainland–island systems, comparable pairs typically show Late Quaternary to Pleistocene splits (e.g., ≈ 0.0172 Mya in the *Hepatica insularis–H. asiatica* complex and ≈ 0.15–2.03 Mya among Korean *Phedimus* endemics), placing the *V. fauriei*–*V. dageletiana* estimate at the extreme recent end of this spectrum (0.021 Mya; 95% HPD 0–0.063 Mya), where limited time for lineage sorting can attenuate cp signals [88,89]. Given the calibration- and prior-sensitivity of absolute dates, we focus on relative ordering rather than precise calendar ages. Regionally, glacial–interglacial climate oscillations and sea-level change structured opportunities for colonization and isolation; in this context, the Holocene-scale split between *V. fauriei* and *V. dageletiana* is consistent with recent colonization or shallow isolation, with incomplete lineage sorting and/or limited gene flow as plausible contributors [90,91]. We therefore use these estimates to highlight the exceptionally recent mainland–island separation of *V. dageletiana* relative to deeper splits elsewhere in the genus. The recent split between *V. fauriei* and *V. dageletiana* suggests limited time for the accumulation of genetic differences, consistent with patterns observed in other recently diverged plant taxa such as *Nymphaea* [92]. We recommend a precautionary approach in view of the very recent split between *V. fauriei* (mainland) and *V. dageletiana* (Ulleungdo): prioritize *in situ* protection of *V. dageletiana* on Ulleungdo; maintain provenance-controlled *ex situ* seed banking for *V. dageletiana*; and defer any translocation or assisted gene flow from *V. fauriei* until genome-wide nuclear evidence indicates low connectivity and introgression risk. Consistent with this precautionary stance, we interpret gene-wise increases in dN/dS as hypothesis-generating signals rather than evidence of adaptation.

Our divergence time estimates provide insights into the selective pressures acting on *Valeriana* species. Among the four species analyzed, *V. jatamansi* diverged approximately 5.394 Mya and exhibited a distinct molecular evolution pattern. Its *psbI* gene showed the highest dN/dS ratio (0.9224), in contrast to the lower values observed in the more recently diverged species. Since *psbI* plays a key role in PSII stabilization and repair, the elevated but subunitary dN/dS is more consistent with lineage-specific modulation or relaxation of selective constraint than with definitive adaptive change, pending orthogonal support (e.g., significant LRT results, site-level signals, or structural/functional evidence). Following this split, *V. officinalis* diverged from the lineage containing *V. fauriei* and *V. dageletiana* approximately 0.735 Mya. This divergence occurred during the middle Pleistocene, yet these species maintained largely similar cp genome structures. No genes showed strong positive selection (dN/dS > 1), but *rps7* and *rpl23* exhibited moderately elevated dN/dS ratios, with *rps7* reaching 0.5388 in *V. jatamansi* and slightly higher values in *V. fauriei* and *V. officinalis*. As ribosomal proteins are essential for translation, these subtle changes could reflect minor, lineage-specific tuning of translational regulation rather than clear evidence of adaptive evolution. The most recent speciation event occurred between *V. fauriei* and *V. dageletiana* approximately 0.021 Mya, a divergence reflected in their highly similar cp genome sequences and minimal dN/dS variation. However, *rpl23* exhibited slightly higher dN/dS values in *V. fauriei* and *V. officinalis*, yet LRTs were non-significant (*p* ≥ 0.79; S8 Table); we therefore interpret this as modest species-level variation within an overall purifying-selection background. Given the recent divergence, limited genetic differentiation between *V. fauriei* and *V. dageletiana* is expected, consistent with their close evolutionary relationship.

In contrast, several photosynthetic genes—such as *psaA*, *psbC*, and *petA*—maintained exceptionally low dN/dS ratios across all four species, reinforcing their strong evolutionary conservation. *psaA*, a PSI subunit involved in electron transport, exhibited values as low as 0.011, underscoring tight functional constraint. Given that all site-model LRTs were non-significant (all *p* > 0.05), these patterns—predominantly low dN/dS in core photosynthetic genes with localized yet subunitary elevations—are best interpreted as a background of strong purifying selection, while allowing for fine-scale, lineage-specific adjustments to be tested in future analyses. Meanwhile, the more recently diverged *V. fauriei*, *V. dageletiana*, and *V. officinalis* share highly similar evolutionary patterns, with only localized yet subunitary elevations in *rps7* and *rpl23*. Accordingly, we treat gene-wise increases in dN/dS ratio as hypothesis-generating rather than evidentiary of adaptive evolution, given non-significant site-model LRTs consistent with minor, lineage-specific modulation of selective constraint. These findings highlight the balance between genetic conservation and lineage-specific molecular evolution in *Valeriana*.

Understanding the selective forces acting on *Valeriana* species provides crucial insights into their adaptive evolution and environmental adaptation. Our findings highlight the significant role that selection pressures play in shaping the evolutionary paths of these species, with each adapting to its unique ecological conditions. These adaptive processes are essential for survival and may contribute to their medicinal potential. By identifying key genes involved in these adaptations, this study lays the foundation for future research exploring how these genetic traits influence the therapeutic properties of *Valeriana*. The close genetic relationship observed between *V. fauriei* and *V. dageletiana* suggests that shared environmental pressures have shaped their similar genomic features, offering important insights into how plants adapt to changing environments. The adaptive evolution of these species reflects a balance between genetic stability and species-specific adaptations that support their ecological roles and potential medicinal value. Understanding the genetic mechanisms behind their adaptive evolution could pave the way for more targeted uses of *Valeriana* in medicine. It also provides a deeper understanding of plant adaptation to environmental stressors, which may inform future conservation efforts.

## Conclusion

In this study, we assembled the complete cp genomes of *V. fauriei* and *V. dageletiana* and confirmed that both share typical angiosperm features, including the standard LSC/SSC/IR organization, 113 unique genes, and comparable GC content. Comparative analyses revealed a consistent expansion of the IR regions relative to *F. cornucopiae*, interspecific differences in the distributions of SSRs and tandem repeats, and sequence-divergence hotspots in *accD*, *rps18*, and the *trnN*–*trnL* region. Selection analyses across four species (*V. fauriei*, *V. dageletiana*, *V. officinalis*, *V. jatamansi*) indicated a predominance of purifying selection. Although *psbI*, *rps7*, and *rpl23* showed relatively elevated dN/dS values, all were < 1, a pattern consistent with lineage-specific relaxation of selective constraint rather than positive selection. Phylogenetic reconstruction recovered *V. fauriei* and *V. dageletiana* as a well-supported sister group within a clade that includes *V. officinalis*, with *V. jatamansi* diverging earlier. Molecular divergence time estimated the divergence of *V. officinalis* at approximately 0.43–1.08 Mya and the split between *V. fauriei* and *V. dageletiana* at 0–0.063 Mya, providing high-resolution evidence for a very recent divergence in the latter. These results show that the cp genomes of *Valeriana* are structurally conserved yet contain species-specific variation sufficient to distinguish taxa, and identify these sequence-divergence hotspots as promising molecular markers for species identification, taxonomy, population genetics, and conservation. Moreover, by integrating structural features, genome-wide variation, selection signals, and molecular-clock information, this study further deepens our understanding of *Valeriana* cp genomes.

## Supporting information

S1 Fig Coverage showing the number of paired-end reads mapped to the complete chloroplast genome of the two *Valeriana* species. LSC, large single-copy region; SSC, small single-copy region; IRa, inverted repeat a; IRb, inverted repeat b.
(PDF)

S2 Fig Codon content and RSCU (Relative synonymous codon usage) values of 20 amino acids and stop codons in *V. fauriei* and *V. dageletiana.* (a) Amino acid frequencies for protein coding sequences. (b) RSCU values for 20 amino acids and stop codons of 79 protein-coding genes.
(PDF)

S3 Fig Codon distribution of protein-coding genes in *Valeriana.* Green and red indicate high and low RSCU (Relative synonymous codon usage) values. Codon pattern analysis was performed using a hierarchical clustering method.
(PDF)

S4 Fig Comparison of the boundaries of the LSC, SSC, and IR regions of three *Valeriana* species and *F. cornucopiae* chloroplast genomes. The number of bp above a gene arrow indicates the distance between the gene and the boundary.
(PDF)

S1 Table Voucher specimen information for chloroplast genomes used in this study. (PDF)

S2 Table Chloroplast genomes from NCBI used for ML, BI, and divergence time phylogenetic analysis. (PDF)

S3 Table Genome assembly data for *Valeriana* chloroplast genomes. (PDF)

S4 Table Raw and trimmed read data. (PDF)

S5 Table Gene according to gene annotation for two *Valeriana* chloroplasts genomes. (PDF)

S6 Table Exon and Intron in *V. fauriei* and *V. dageletiana* chloroplast genomes. The numbers in the Exon and Intron columns represent the base pair lengths in the chloroplast genomes of *V. fauriei* and *V. dageletiana*. Intron II in *rps12* is present, while Intron I is absent.
(PDF)

S7 Table dN/dS values estimated under the M1a (neutral) and M2a (positive selection) models in comparison with *F. cornucopiae.* The M1a model assumes a neutral evolutionary process with dN/dS ratio constrained to ≤ 1, while the M2a model allows for the presence of positively selected sites (dN/dS ratio > 1).
(PDF)

S8 Table Log-likelihood values of the M1a and M2a models and likelihood ratio test (LRT) results. (PDF)
