# Supplementary material for: Chloroplast genome comparison of Valeriana species with sequence variation, selective pressure, and divergence analysis
Source: PLoS One. 2026 Mar 17;21(3):e0344868. doi: 10.1371/journal.pone.0344868 (PMC12994825; doi:10.1371/journal.pone.0344868)
Supplement: S1 Fig — LSC, large single-copy region; SSC, small single-copy region; IRa, inverted repeat a; IRb, inverted repeat b. (PDF) [file pone.0344868.s001.pdf]

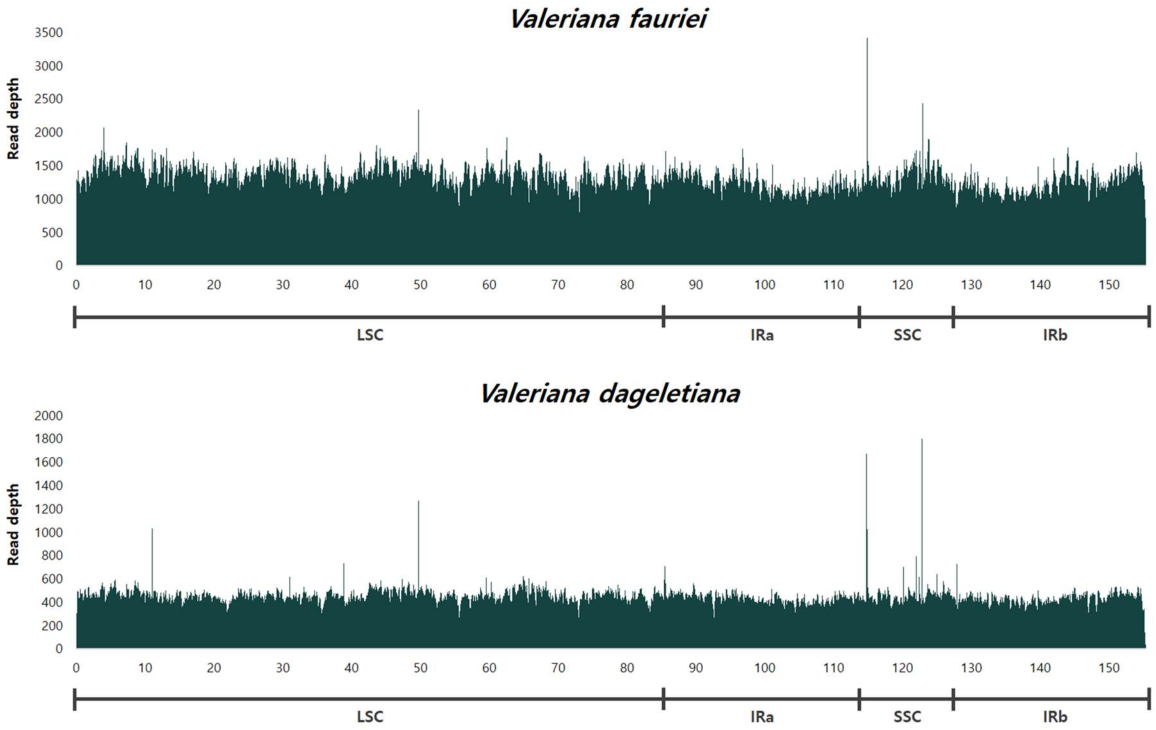

S1 Fig. Coverage showing the number of paired-end reads mapped to the complete chloroplast genome of the two *Valeriana* species. LSC, large single-copy region; SSC, small single-copy region; IRa, inverted repeat a; IRb, inverted repeat b.
