# Supplementary material for: Chloroplast genome comparison of Valeriana species with sequence variation, selective pressure, and divergence analysis
Source: PLoS One. 2026 Mar 17;21(3):e0344868. doi: 10.1371/journal.pone.0344868 (PMC12994825; doi:10.1371/journal.pone.0344868)
Supplement: S3 Fig — Green and red indicate high and low RSCU (Relative synonymous codon usage) values. Codon pattern analysis was performed using a hierarchical clustering method. (PDF) [file pone.0344868.s003.pdf]

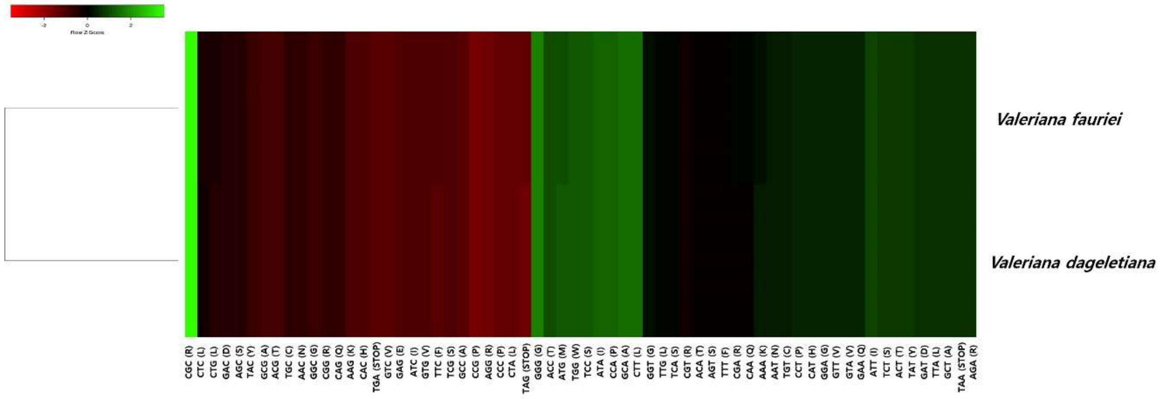

S3 Fig. Codon distribution of protein-coding genes in *Valeriana*. Green and red indicate high and low RSCU (Relative Synonymous Codon Usage) values. Codon pattern analysis was performed using a hierarchical clustering method.
