# Supplementary material for: Chloroplast genome comparison of Valeriana species with sequence variation, selective pressure, and divergence analysis
Source: PLoS One. 2026 Mar 17;21(3):e0344868. doi: 10.1371/journal.pone.0344868 (PMC12994825; doi:10.1371/journal.pone.0344868)
Supplement: S4 Fig — The number of bp above a gene arrow indicates the distance between the gene and the boundary. (PDF) [file pone.0344868.s004.pdf]

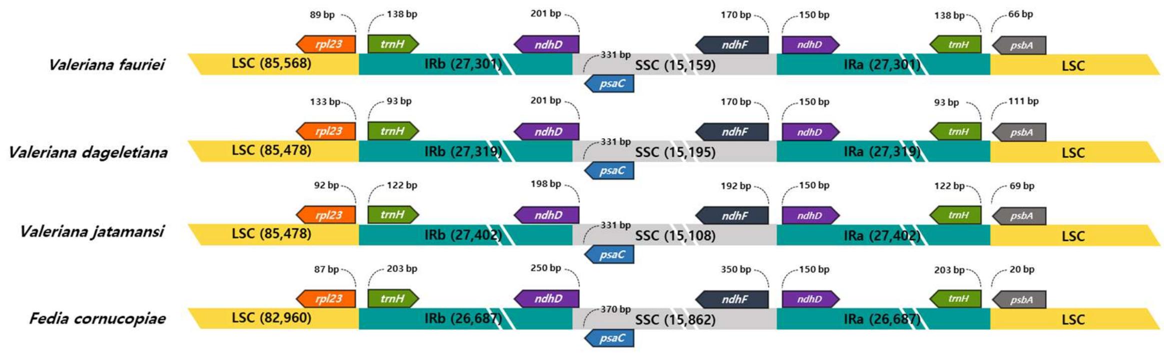

S4 Fig. Comparison of the boundaries of the LSC, SSC, and IR regions of three *Valeriana* species and *Fedia cornucopiae* chloroplast genomes. The number of bp above a gene arrow indicates the distance between the gene and the boundary.
