# Supplementary material for: Chloroplast genome comparison of Valeriana species with sequence variation, selective pressure, and divergence analysis
Source: PLoS One. 2026 Mar 17;21(3):e0344868. doi: 10.1371/journal.pone.0344868 (PMC12994825; doi:10.1371/journal.pone.0344868)
Supplement: S1 Table — (PDF) [file pone.0344868.s005.pdf]

S1 Table. Voucher specimen information for chloroplast genomes used in this study.

Species	Collection information	Coordinates	Voucher number
V. fauriei	Soi-myeon, Eumseong-gun,<br>Chungcheongbuk-do, Repulic of Korea	36°56'37.9"N<br>127°45'11.9"E	Cultivar
V. dageletiana	Seo-myeon, Ulleung-gun,<br>Gyeongsangbuk-do, Republic of Korea	37°28'06.3"N<br>130°50'33.4"E	HNIBRVP18373
