# Supplementary material for: Chloroplast genome comparison of Valeriana species with sequence variation, selective pressure, and divergence analysis
Source: PLoS One. 2026 Mar 17;21(3):e0344868. doi: 10.1371/journal.pone.0344868 (PMC12994825; doi:10.1371/journal.pone.0344868)
Supplement: S2 Table — (PDF) [file pone.0344868.s006.pdf]

S2 Table. Chloroplast genomes from NCBI used for ML, BI, and divergence time phylogenetic analysis.

No.	Family	Subfamily	Taxon	Genbank accession number
1	Caprifoliaceae	Valerianoideae	*Valeriana jatamansi*	NC_067975
2	Caprifoliaceae	Valerianoideae	*Valeriana officinalis*	NC_045052
3	Caprifoliaceae	Valerianoideae	*Fedia cornucopiae*	NC_065839
4	Caprifoliaceae	Dipsaceae	*Dipsacus asper*	NC_039748
5	Caprifoliaceae	Dipsaceae	*Dipsacus japonicus*	NC_039668
6	Caprifoliaceae	Scabioseae	*Scabiosa comosa*	NC_065840
7	Caprifoliaceae	Scabioseae	*Scabiosa tschiliensis*	NC_045050
