# Supplementary material for: Chloroplast genome comparison of Valeriana species with sequence variation, selective pressure, and divergence analysis
Source: PLoS One. 2026 Mar 17;21(3):e0344868. doi: 10.1371/journal.pone.0344868 (PMC12994825; doi:10.1371/journal.pone.0344868)
Supplement: S3 Table — (PDF) [file pone.0344868.s007.pdf]

S3 Table. Genome assembly data for *Valeriana* chloroplast genomes.

Scientific name	Aligned reads (#)	Coverage (x)	Cp genome length (bp)
V. fauriei	1,460,714	1,328	155,329
V. dageletiana	528,511	453	155,311
