# Supplementary material for: Chloroplast genome comparison of Valeriana species with sequence variation, selective pressure, and divergence analysis
Source: PLoS One. 2026 Mar 17;21(3):e0344868. doi: 10.1371/journal.pone.0344868 (PMC12994825; doi:10.1371/journal.pone.0344868)
Supplement: S4 Table — (PDF) [file pone.0344868.s008.pdf]

S4 Table. Raw and trimmed read data.

Scientific name	Input reads	Trimmed reads		Raw bases	Trimmed bases	Sequencing platform
V. fauriei	84,442,176	37,053,518	43.28%	12,750,768,576	5,518,786,653	Novaseq 6000
V. dageletiana	81,539,612	65,377,580	79.22%	12,312,481,412	9,753,234,382	Novaseq 6000
