# Supplementary material for: Chloroplast genome comparison of Valeriana species with sequence variation, selective pressure, and divergence analysis
Source: PLoS One. 2026 Mar 17;21(3):e0344868. doi: 10.1371/journal.pone.0344868 (PMC12994825; doi:10.1371/journal.pone.0344868)
Supplement: S5 Table — (PDF) [file pone.0344868.s009.pdf]

S5 Table. Gene according to gene annotation for two *Valeriana* chloroplasts genomes.

Group of genes	Name of genes
Photosystem I	*psaA*, *B*, *C*, *I*, *J*, *pafI*[2)], *pafII*
Photosystem II	*psbA*, *B*, *C*, *D*, *E*, *F*, *H*, *I*, *J*, *K*, *L*, *M*, *T*, *Z*, *pbf1*
Cytochrome b6/f	*petA*, *B*[1)], *G*, *D*[1)], *L*, *N*
ATP synthase	*atpA*, *B*, *E*, *F*[1)], *H*, *I*
Rubisco	*rbcL*
NADH oxidoreductase	*ndhA*[1)], *B*[1)3)], *C*, *D*[3)], *E*, *F*, *G*, *H*, *I*, *J*, *K*
Large subunit ribosomal proteins	*rpl2*[1)], *14*, *16*[1)], *20*, *22*, *23*, *32*, *33*, *36*
Small subunit ribosomal proteins	*rps2*, *3*, *4*, *7*[3)], *8*, *11*, *12*[2)3)4)], *14*, *15*, *16*[1)], *18*, *19*
RNA polymerase	*rpoA*, *B*, *C1*[1)], *C2*
Unknown function protein coding gene	*ycf1*, *ycf2*[3)]
Other genes	*accD*, *ccsA*[3)], *cemA*, *clpP1*[2)], *infA*, *matK*
Ribosomal RNAs	*rrn4.5*[3)], *rrn5*[3)], *rrn16*[3)], *rrn23*[3)]
Transfer RNAs	*trnA-UGC*[1)3)], *trnC-GCA*, *trnD-GUC*, *trnE-UUC*, *trnF-GAA*, *trnfM-CAU*, *trnG-GCC*, *trnH-GUG*, *trnl-CAU*[3)], *trnl-GAU*[1)3)], *trnK-UUU*[1)], *trnL-CAA*[3)], *trnL-UAA*[1)], *trnL-UAG*, *trnM-CAU*[3)], *trnN-GUU*[3)], *trnP-UGG*, *trnQ-UUG*[3)], *trnR-ACG*[3)], *trnR-UCU*, *trnS-CGA*, *trnS-GCU*, *trnS-GGA*, *trnS-UGA*, *trnT-GGU*, *trnT-UGU*, *trnV-GAC*[3)], *trnV-UAC*[1)], *trnW-CCA*, *trnY-GUA*

1) Gene containing a single intron, 2) Gene containing two introns, 3) Two gene copies in IRs, 4) Trans-splicing gene
