# Supplementary material for: Chloroplast genome comparison of Valeriana species with sequence variation, selective pressure, and divergence analysis
Source: PLoS One. 2026 Mar 17;21(3):e0344868. doi: 10.1371/journal.pone.0344868 (PMC12994825; doi:10.1371/journal.pone.0344868)
Supplement: S6 Table — The numbers in the Exon and Intron columns represent the base pair lengths in the chloroplast genomes of V. fauriei and V. dageletiana. Intron II in rps12 is present, while Intron I is absent. (PDF) [file pone.0344868.s010.pdf]

S6 Table. Exon and Intron in *V. fauriei* and *V. dageletiana* chloroplast genomes. The numbers in the Exon and Intron columns represent the base pair lengths in the chloroplast genomes of *V. fauriei* and *V. dageletiana*. Intron II in *rps12* is present, while Intron I is absent.

V. fauriei	Gene	Region	Exon I	Intron I	Exon II	Intron II	Exon III
1	*trnK-UUU*	LSC	37	2492	35		
2	*rps16*	LSC	40	832	218		
3	*atpF*	LSC	144	708	411		
4	*rpoC1*	LSC	468	760	1623		
5	*pafI*	LSC	124	732	228	747	155
6	*trnL-UAA*	LSC	37	494	50		
7	*trnV-UAC*	LSC	39	574	37		
8	*rps12*	LSC, IR	114	-	232	540	26
9	*clpP1*	LSC	70	772	293	608	246
10	*petB*	LSC	6	775	642		
11	*petD*	LSC	8	741	475		
12	*rpl16*	LSC	9	1138	399		
13	*rpl2*	LSC	391	654	434		
14	*ndhB*	IR	777	678	756		
15	*trnI-GAU*	IR	37	948	35		
16	*trnA-UGC*	IR	38	806	35		
17	*ndhA*	SSC	552	1072	540		

V. dageletiana	Gene	Region	Exon I	Intron I	Exon II	Intron II	Exon III
1	*trnK-UUU*	LSC	37	2492	36		
2	*rps16*	LSC	40	832	218		
3	*atpF*	LSC	144	708	411		
4	*rpoC1*	LSC	468	760	1623		
5	*pafI*	LSC	124	732	228	747	155
6	*trnL-UAA*	LSC	37	494	50		
7	*trnV-UAC*	LSC	39	574	37		
8	*rps12*	LSC, IR	114	-	232	654	26
9	*clpP1*	LSC	70	772	293	608	246
10	*petB*	LSC	6	775	642		
11	*petD*	LSC	8	741	475		
12	*rpl16*	LSC	9	1138	399		
13	*rpl2*	LSC	391	654	434		
14	*ndhB*	IR	777	678	756		
15	*trnI-GAU*	IR	37	948	35		
16	*trnA-UGC*	IR	38	806	35		
17	*ndhA*	SSC	552	1072	540		
