# Supplementary material for: Chloroplast genome comparison of Valeriana species with sequence variation, selective pressure, and divergence analysis
Source: PLoS One. 2026 Mar 17;21(3):e0344868. doi: 10.1371/journal.pone.0344868 (PMC12994825; doi:10.1371/journal.pone.0344868)
Supplement: S7 Table — The M1a model assumes a neutral evolutionary process with dN/dS ratio constrained to ≤ 1, while the M2a model allows for the presence of positively selected sites (dN/dS ratio > 1). (PDF) [file pone.0344868.s011.pdf]

S8 Table. Log-likelihood values of the M1a and M2a models and likelihood ratio test (LRT) results. Log-likelihood (InL) values for each gene were estimated under the M1a (neutral) model and the M2a (positive selection) model, based on dN/dS values derived from comparisons between *Fedia cornucopiae* and four *Valeriana* species. A LRT was performed to compare the two models. If p-value > 0.05, the M1a model is considered more appropriate.

	F. cornucopiae* vs *V. fauriei				***F. cornucopiae* vs *V. dageletiana***			
Gene	M1a	M2a	LRT	p_value	M1a	M2a	LRT	p_value
atpA	-2217.43	-2217.43	0.000236	0.999882	-2217.43	-2217.43	0.000236	0.999882
atpB	-2215.96	-2215.96	5.8E-05	0.999971	-2215.96	-2215.96	5.8E-05	0.999971
atpE	-562.548	-562.548	8E-06	0.999996	-562.548	-562.548	8E-06	0.999996
atpF	-841.279	-841.279	2E-06	0.999999	-841.279	-841.279	2E-06	0.999999
atpI	-1062.76	-1062.73	0.062396	0.969284	-1062.76	-1062.73	0.062396	0.969284
cemA	-1032.37	-1032.37	2E-06	0.999999	-1032.37	-1032.37	2E-06	0.999999
matK	-2450.39	-2449.49	1.792678	0.408061	-2450.39	-2449.49	1.792678	0.408061
ndhB	-2061.06	-2061.06	-8E-06	1	-2061.06	-2061.06	0	1
ndhC	-519.149	-518.963	0.371672	0.83041	-519.149	-518.963	0.371672	0.83041
ndhE	-431.413	-431.413	2.4E-05	0.999988	-431.413	-431.413	2.4E-05	0.999988
ndhG	-806.06	-806.049	0.02121	0.989451	-806.049	-806.049	0	1
ndhH	-1763.93	-1763.93	0.000142	0.999929	-1763.93	-1763.93	0.000142	0.999929
ndhI	-771.721	-771.721	6.6E-05	0.999967	-771.721	-771.721	6.6E-05	0.999967
ndhJ	-718.706	-716.088	5.236332	0.072937	-718.706	-716.088	5.236334	0.072936
pafII	-854.871	-854.871	7.2E-05	0.999964	-854.871	-854.871	7.2E-05	0.999964
pafI	-718.237	-718.237	1.2E-05	0.999994	-718.237	-718.237	1.2E-05	0.999994
petA	-1431.37	-1431.37	0	1	-1431.37	-1431.37	0	1
petB	-919.031	-918.829	0.40412	0.817046	-919.031	-918.829	0.40412	0.817046
psaA	-3262.78	-3262.78	6E-06	0.999997	-3262.78	-3262.78	6E-06	0.999997
psaB	-3157.04	-3154.78	4.514836	0.10462	-3157.04	-3154.78	4.514836	0.10462
psaJ	-191.667	-191.574	0.186584	0.910927	-191.667	-191.574	0.186584	0.910927
psbB	-2187.2	-2187.2	0.00012	0.99994	-2187.2	-2187.2	0.00012	0.99994
psbC	-2068.45	-2068.45	0.000348	0.999826	-2068.45	-2068.45	0.000348	0.999826
psbH	-317.179	-317.179	4.4E-05	0.999978	-317.179	-317.179	4.4E-05	0.999978
psbI	-157.573	-157.565	0.015096	0.99248	-157.573	-157.565	0.015096	0.99248
psbK	-283.229	-283.229	9.4E-05	0.999953	-283.229	-283.229	9.4E-05	0.999953
rbcL	-2064.03	-2064.03	9.2E-05	0.999954	-2064.03	-2064.03	9.2E-05	0.999954
rpl14	-541.828	-540.068	3.51895	0.172135	-541.828	-540.068	3.518948	0.172135
rpl16	-594.843	-594.843	2.4E-05	0.999988	-594.843	-594.843	2.4E-05	0.999988
rpl22	-729.185	-729.185	2.2E-05	0.999989	-729.185	-729.185	2.2E-05	0.999989
rpl23	-450.768	-450.537	0.46193	0.793767	-450.768	-450.537	0.46193	0.793767
rpl2	-1257.01	-1257.01	5.8E-05	0.999971	-1257.01	-1257.01	5.8E-05	0.999971
rpl33	-312.235	-312.235	0	1	-312.235	-312.235	0	1
rpoA	-1532.67	-1532.67	2.6E-05	0.999987	-1532.67	-1532.67	2.6E-05	0.999987
rps11	-619.403	-619.403	3.6E-05	0.999982	-619.403	-619.403	3.6E-05	0.999982
rps14	-451.87	-451.87	8E-06	0.999996	-451.87	-451.87	8E-06	0.999996
rps2	-1094.87	-1094.87	9.6E-05	0.999952	-1094.87	-1094.87	9.6E-05	0.999952
rps4	-908.322	-908.322	5.6E-05	0.999972	-908.322	-908.322	5.6E-05	0.999972
rps7	-617.212	-617.225	-0.02483	1	-617.212	-617.212	0	1
rps8	-645.204	-645.204	2.2E-05	0.999989	-645.204	-645.204	2.2E-05	0.999989

	F. cornucopiae vs *V. officinalis*				*F. cornucopiae vs V. jatamansi*			
Gene	M1a	M2a	LRT	p_value	M1a	M2a	LRT	p_value
atpA	-2217.43	-2217.43	0.000236	0.999882	-2217.43	-2217.43	0.000236	0.999882
atpB	-2215.96	-2215.96	5.8E-05	0.999971	-2215.96	-2215.96	5.8E-05	0.999971
atpE	-562.548	-562.548	8E-06	0.999996	-562.548	-562.548	8E-06	0.999996
atpF	-841.279	-841.279	2E-06	0.999999	-841.279	-841.279	2E-06	0.999999
atpI	-1062.76	-1062.73	0.062396	0.969284	-1062.76	-1062.73	0.062396	0.969284
cemA	-1032.37	-1032.37	2E-06	0.999999	-1032.37	-1032.37	2E-06	0.999999
matK	-2446.39	-2445.73	1.337444	0.512363	-2450.39	-2449.49	1.792678	0.408061
ndhB	-2061.06	-2061.06	0	1	-2061.06	-2061.06	-8E-06	1
ndhC	-519.149	-518.963	0.371672	0.83041	-519.149	-518.963	0.371672	0.83041
ndhE	-431.413	-431.413	2.4E-05	0.999988	-431.413	-431.413	2.4E-05	0.999988
ndhG	-800.283	-800.188	0.190436	0.909175	-806.06	-806.049	0.02121	0.989451
ndhH	-1764.72	-1764.72	0.00017	0.999915	-1763.93	-1763.93	0.000142	0.999929
ndhI	-767.87	-767.87	5.6E-05	0.999972	-771.721	-771.721	6.6E-05	0.999967
ndhJ	-718.706	-716.088	5.236334	0.072936	-718.706	-716.088	5.236332	0.072937
pafII	-854.871	-854.871	7.2E-05	0.999964	-854.871	-854.871	7.2E-05	0.999964
pafI	-718.237	-718.237	1.2E-05	0.999994	-718.237	-718.237	1.2E-05	0.999994
petA	-1434.21	-1434.21	0	1	-1431.37	-1431.37	0	1
petB	-919.031	-918.829	0.40412	0.817046	-919.031	-918.829	0.40412	0.817046
psaA	-3262.78	-3262.78	6E-06	0.999997	-3262.78	-3262.78	6E-06	0.999997
psaB	-3157.04	-3154.78	4.514836	0.10462	-3157.04	-3154.78	4.514836	0.10462
psaJ	-191.667	-191.574	0.186584	0.910927	-191.667	-191.574	0.186584	0.910927
psbB	-2184.66	-2184.66	0.000112	0.999944	-2187.2	-2187.2	0.00012	0.99994
psbC	-2068.45	-2068.45	0.000348	0.999826	-2068.45	-2068.45	0.000348	0.999826
psbH	-317.179	-317.179	4.4E-05	0.999978	-317.179	-317.179	4.4E-05	0.999978
psbI	-157.573	-157.565	0.015096	0.99248	-157.573	-157.565	0.015096	0.99248
psbK	-283.229	-283.229	9.4E-05	0.999953	-283.229	-283.229	9.4E-05	0.999953
rbcL	-2064.03	-2064.03	9.2E-05	0.999954	-2064.03	-2064.03	9.2E-05	0.999954
rpl14	-541.828	-540.068	3.518948	0.172135	-541.828	-540.068	3.51895	0.172135
rpl16	-594.843	-594.843	2.4E-05	0.999988	-594.843	-594.843	2.4E-05	0.999988
rpl22	-741.196	-741.196	4E-06	0.999998	-729.185	-729.185	2.2E-05	0.999989
rpl23	-450.768	-450.537	0.46193	0.793767	-450.768	-450.537	0.46193	0.793767
rpl2	-1262.73	-1262.73	5.6E-05	0.999972	-1257.01	-1257.01	5.8E-05	0.999971
rpl33	-312.235	-312.235	0	1	-312.235	-312.235	0	1
rpoA	-1536.13	-1536.13	1.2E-05	0.999994	-1532.67	-1532.67	2.6E-05	0.999987
rps11	-619.403	-619.403	3.6E-05	0.999982	-619.403	-619.403	3.6E-05	0.999982
rps14	-451.87	-451.87	8E-06	0.999996	-451.87	-451.87	8E-06	0.999996
rps2	-1094.87	-1094.87	9.6E-05	0.999952	-1094.87	-1094.87	9.6E-05	0.999952
rps4	-913.8	-913.8	5.6E-05	0.999972	-908.322	-908.322	5.6E-05	0.999972
rps7	-617.212	-617.212	0	1	-617.212	-617.225	-0.02483	1
rps8	-645.204	-645.204	2.2E-05	0.999989	-645.204	-645.204	2.2E-05	0.999989
