# Supplementary material for: Chloroplast genome comparison of Valeriana species with sequence variation, selective pressure, and divergence analysis
Source: PLoS One. 2026 Mar 17;21(3):e0344868. doi: 10.1371/journal.pone.0344868 (PMC12994825; doi:10.1371/journal.pone.0344868)
Supplement: S2 Fig — (a) Amino acid frequencies for protein coding sequences. (b) RSCU values for 20 amino acids and stop codons of 79 protein-coding genes. (PDF) [file pone.0344868.s002.pdf]

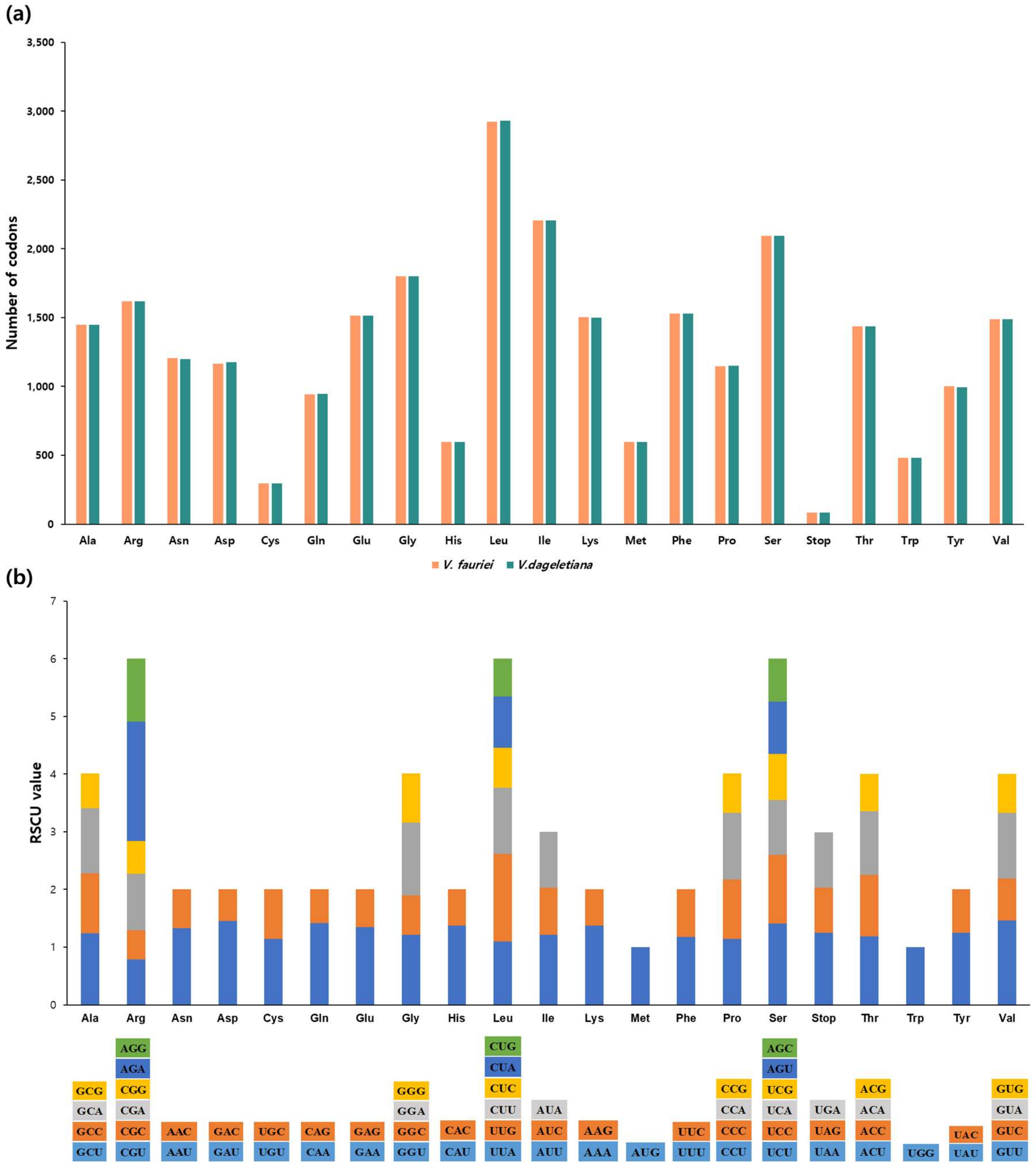

S2 Fig. Codon content and RSCU (Relative Synonymous Codon Usage) values of 20 amino acids and stop codons in *V. fauriei* and *V. dageletiana*. (a) Amino acid frequencies for protein coding sequences. (b) RSCU values for 20 amino acids and stop codons of 79 protein-coding genes.
